# Post-cardiac Arrest Leukocytosis Mimicking Acute Monocytic Leukemia

**DOI:** 10.7759/cureus.29508

**Published:** 2022-09-23

**Authors:** Navkirat Kahlon, Sishir Doddi, Ziad Abuhelwa, Lauren Stanoszek, Danae Hamouda

**Affiliations:** 1 Hematology and Medical Oncology, The University of Toledo Medical Center, Toledo, USA; 2 Medicine, The University of Toledo College of Medicine and Life Sciences, Toledo, USA; 3 Internal Medicine, The University of Toledo, Toledo, USA; 4 Pathology, The University of Toledo, Toledo, USA; 5 Hematology and Medical Oncology, The University of Toledo, Toledo, USA

**Keywords:** abnormal peripheral blood smear, mimicking leukemia, post-cardiac arrest, leukemoid reaction, reactive leukocytosis

## Abstract

Leukocytosis is defined by an increased WBC count in the peripheral blood. This can be caused by many pathologies from benign conditions such as stress, infection, and inflammation or malignant origins such as leukemia. Although leukocytosis is regularly encountered clinically and has many etiologies making a definitive diagnosis, at times, may be difficult. A case of severe leukocytosis requires careful consideration of symptoms and confirmation with serial complete blood count (CBC) testing before pursuing further invasive testing such as bone marrow biopsy. Here, we report the case of a 78-year-old male patient who, after a cardiac arrest, presented with reactive hyperleukocytosis mimicking acute monocytic leukemia.

## Introduction

Leukocytosis, an increase in WBC count, is a commonly encountered laboratory finding. For most healthy adults, normal WBC levels typically range from 4,500 to 11,000 per microliter of blood. Leukocytosis is defined as a WBC count higher than 11,000 per microliter of blood. This finding has wide differential diagnoses ranging from benign causes, such as inflammation, infection, stress, or medication use, to malignant causes, such as chronic and acute leukemia. Stems cells in the bone marrow produce megakaryoblasts, lymphoblasts, and myeloblasts, which eventually mature into WBCs. After maturation, 80-90% of WBCs remain in the bone marrow for storage. Of the remaining 10-20%, the peripheral blood system has a circulating pool of only 2-3% leukocytes, and the remainder are stored in the spleen or on the walls of blood vessels [1]. Leukocytosis can be due to the release of these stored WBCs (reactive) or excess production. The bone marrow increases the production of WBCs in response to inflammation, infection, and physical and mental stress. Stress can cause the release of WBCs stored in the bone marrow, which could present as reactive leukocytosis [2]. Routine medications, such as corticosteroids, lithium, and beta-agonists, cause leukocytosis as well. The most common malignant causes of leukocytosis are bone marrow disorders such as acute leukemia, chronic leukemia, and myeloproliferative disorders [3]. 

Hyperleukocytosis is rare and defined as a WBC count above 50x10^9 per liter (/L). Hyperleukocytosis supports the consideration of workup for acute leukemia [4]. The first step for further evaluation of hyperleukocytosis is obtaining a peripheral smear. A bone marrow biopsy is typically obtained to confirm the diagnosis if a peripheral smear is suspicious for acute leukemia. We are reporting a case of hyperleukocytosis in the setting of cardiac arrest where initial laboratory testing was highly suspicious for acute monocytic leukemia. Acute monocytic leukemia is a subtype of acute myeloid leukemia characterized by 80% of leukemic cells of monocytic lineage, that is, monoblasts, promonocytes, and monocytes. Monocytes are leukocytes that circulate in the blood and eventually migrate into tissues to develop into macrophages. These cells play a vital role in the innate immune system and are implicated in the body’s physiological defense against pathogens and the inflammatory response. Acute monocytic leukemia has a higher association with leukostasis than other acute leukemias. Leukostasis can result in hyper viscosity and clinical presentation can be altered mental status, focal neurologic deficits, deep venous thrombosis, avascular bone necrosis, sudden cardiac arrest, splenic rupture, acute kidney injury, or retinopathy [5,6]. 

Clinically, it is crucial to consider the broad differential diagnosis before pursuing further invasive diagnostic procedures when assessing leukocytosis [7]. We report the case of severe leukocytosis post-cardiac arrest with a preliminary clinical picture mimicking acute monocytic leukemia. This severe leukocytosis eventually turned out to be reactive in nature. This case highlights the importance of repeating abnormal laboratory studies to ensure persistence before relaying this information to the patient or family and proceeding with further invasive procedures.

## Case presentation

After cardiac arrest, a 78-year-old man presented to the ED due to pulseless electrical activity and unknown downtime. He was found unresponsive by a nurse in the skilled nursing facility. Cardiopulmonary resuscitation and advanced care life support protocol were initiated at the facility. He achieved a return of spontaneous circulation after 20 minutes of resuscitation measures. Upon arrival at the ED, he was unresponsive with intermittent seizures, so he was immediately intubated and started on mechanical ventilation. 

One day prior to his presentation, he was at his baseline clinical condition per visiting family members and staff at the skilled nursing facility. He was lucid and needed assistance with daily tasks at baseline. He had no prior history of myocardial infarction. Although he had past medical history of chronic obstructive pulmonary disease due to emphysema, type 2 diabetes mellitus, essential hypertension, paroxysmal atrial fibrillation, chronic kidney disease stage IIIA, and monoclonal gammopathy of uncertain significance.

Laboratory investigations on initial presentation showed a WBC count of 111×109/liter (L) (normal 4-10.6×109/L) with a differential as the following: monocytes 65%, lymphocytes 17%, neutrophils 7%, and immature granulocytes 6.9%. Hemoglobin was 6.6 gram (g)/deciliter (dL) (normal 13.0-17.0 g/dl) and platelet count was 82×109/L (normal 13.0-17.0×109/L). Two weeks prior to presentation, complete blood count (CBC) showed a WBC count of 12.6×109/L (monocytes 19.5%, lymphocytes 46.3%, neutrophils 33.3%), hemoglobin 10 mg/dL, and platelet count 171×109/L. Other laboratory studies are summarized in Table 1.

**Table 1:** Laboratory results of the patient. BUN: Blood urea nitrogen; mg/dl: Milligrams per decilitre; AST: Aspartate transaminase; U/L: Units/liter; ALT: Alanine transaminase; mmol/L: Millimoles per liter; g/dl: Grams per decilitre; ng/mL: Nanograms per milliliter; BNP: Brain natriuretic peptide; pg/ml: Picograms per milliliter; PT: Prothrombin time; sec: Seconds; INR: International normalized ratio; PTT: Partial prothrombin time.

Laboratory study	Patient results	Normal range
BUN	22 mg/dL	5-23 mg/dL
Creatinine	1.53 mg/dl	0.60-1.30 mg/dL
AST	89 U/L	0-41 U/L
ALT	35 U/L	0-40 U/L
Lactate	11.3 mmol/L:	0.5-2.2 mmol/L
Total protein	5 g/dL	6-8.3 g/dL
Albumin	2.4 g/dL	3.5-5.7 g/dL
Troponin	0.07 ng/mL	< 0.04 ng/mL)
BNP	65 pg/ml	0-100 pg/ml
PT	19.7 sec	9.5-12.6 sec
INR	1.65	0.9-1.2
PTT	63.8 sec	26-37 sec

**Figure 1:**
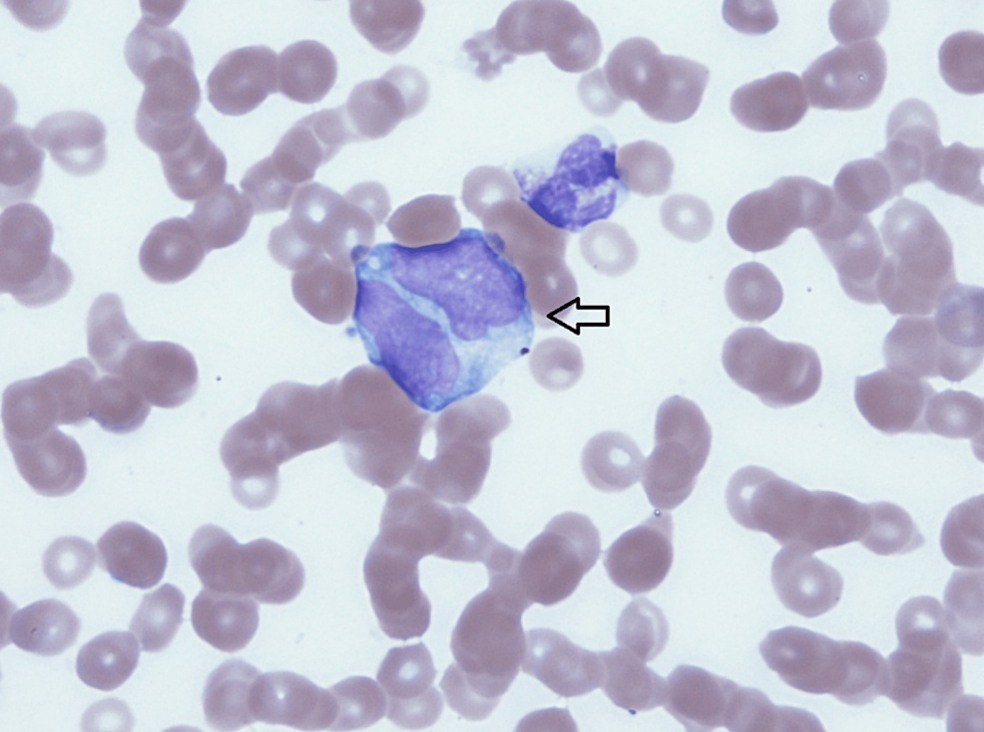
Patient’s peripheral blood smear with 100x objective lens. Immature monocytes seen in patient’s peripheral blood.

**Figure 2:**
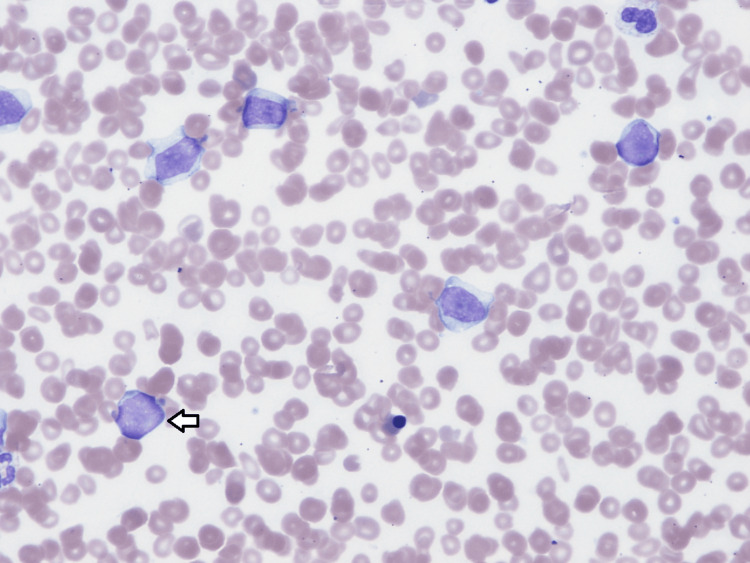
Patient’s peripheral blood smear with 50x objective lens. Myeloblasts and immature monocytes seen in patient’s peripheral blood.

Imaging studies done in the ED included an electrocardiogram that showed sinus tachycardia without ST or T wave changes. Echocardiography showed preserved left ventricular systolic function with no regional wall motion abnormalities. Chest CT angiography was negative for pulmonary embolism; however, it showed multiple ribs and sternal fractures in addition to right lower lobe consolidation. These fractures were most likely secondary to CPR. CT imaging of the abdomen, pelvis, and brain was unremarkable. Based on the clinical picture, consideration of acute leukemia resulting in leukostasis and, thus, sudden cardiac arrest was among differentials. A peripheral blood smear showed atypical monocytic cells (immature monocytes and myeloblasts), which made the working diagnosis of acute monocytic leukemia more likely. The peripheral blood smear is shown in Figures 1-2.

A decision to repeat the CBC was made prior to the discussion regarding the need for a bone marrow biopsy and a working diagnosis of acute leukemia. Repeated CBC nine hours later showed a significant decrease in WBC count to 11.5×109/L with 45.6% monocytes. The cardiac arrest was eventually attributed to aspiration pneumonia. He continued to receive post-cardiac arrest care in the ICU. He received IV fluids, vasopressors, packed RBC transfusion, and broad-spectrum antibiotics. However, he continued to remain unresponsive and did not follow commands. Care was withdrawn per the family's wish, given the patient's poor prognosis, primarily secondary to underlying anoxic brain injury. Comfort care measures were started, and he expired two days after the initial presentation. Bone marrow biopsy thus could not be obtained.

## Discussion

Leukocytosis is a commonly encountered finding during clinical practice. It is essential to distinguish between malignant and non-malignant causes of leukocytosis. Our patient, on initial presentation, had severe leukocytosis with monocytic predominance in CBC and myeloblasts and premature monocytes in the peripheral blood smear. Both these results were consistent with an underlying acute monocytic leukemia. In certain situations, hyperleukocytosis due to an underlying malignancy can be a precipitating factor for cardiac arrest. This is more common with acute monocytic leukemia than other acute leukemias [6]. Thus, the laboratory studies and the clinical picture, including an initial presentation with cardiac arrest (thought to be a result of leukostasis), were highly suggestive of acute monocytic leukemia in our case. The preliminary diagnosis of leukemia was communicated to the primary team by the pathology team. However, a repeat blood workup nine hours later showed a dramatic decrease in WBC count to 11.5×109/L with 45.6% monocytes, making underlying acute leukemia a less likely diagnosis. The half-life of circulating WBCs is seven hours which could explain the drop in WBC count on repeat CBC. The severe leukocytosis was therefore deemed to be reactive in nature, secondary to the stress from cardiac arrest [8]. This disappearance of reactive leukocytes led to near-normalization of WBC count. 

Post-cardiac arrest leukocytosis is a known phenomenon resulting from endogenous alarm signals and sterile inflammation of body tissues [9]. A study found that after cardiac arrest and during post-cardiac arrest syndrome (PCAS), there is a change in monocytes' pattern recognition receptor cell signaling pathway. During the cardiac arrest and cardiopulmonary resuscitation, danger-associated molecular pattern (DAMPs) molecules are released and are recognized by pattern recognition receptors, specifically toll-like receptors (TLRs) on monocytes [9]. A review article by Jimenez-Dalmaroni et al. found that TLRs play a role in increasing the body's pro-inflammatory response, possibly leading to hyperleukocytosis [9]. An additional study found that the WBC count of patients after cardiac arrest ranges from 9.0-21.7x10^9/L [10]. Our case was unique in the magnitude of WBC elevation and peripheral smear mimicking the acute leukemia picture.

Hyperleukocytosis, in this case, was most likely a bone marrow reaction due to acute stressor (cardiac arrest) rather than acute leukemia. However, we acknowledge that an undiagnosed underlying chronic myelomonocytic leukemia (CMML) is possible given persistent chronic monocytosis on CBC. Also, it could have been responsible for monocyte predominance in peripheral blood in the setting of acute stress. Bone marrow biopsy and aspirate are needed to confirm this diagnosis of CMML; unfortunately, a bone marrow biopsy could not be obtained due to the patient's death two days post-cardiac arrest and presentation. Our case highlights the importance of repeating abnormal laboratory testing before proceeding with further invasive testing or evaluation. Particularly, repeating a CBC after a significant stress event such as cardiac arrest is important to confirm persistent leukocytosis.

## Conclusions

Severe leukocytosis can be due to different causes, including malignant and non-malignant. After cardiac arrest, severe reactive leukocytosis can be seen in rare cases, and clinical pictures (CBC, peripheral smear, and initial clinical presentation) can mimic acute leukemia. Therefore, we recommend repeating the CBC a few hours later before pursuing further invasive testing for acute leukemia, such as bone marrow biopsy. 
